# Supplementary material for: MetaRibo-Seq measures translation in microbiomes
Source: Nat Commun. 2020 Jun 29;11:3268. doi: 10.1038/s41467-020-17081-z (PMC7324362; doi:10.1038/s41467-020-17081-z)
Supplement: Supplementary file 10 — Supplementary Data 7 [file 41467_2020_17081_MOESM10_ESM.zip › File2/Confidence_High_Taxonomy/428466_out.krona.html]

Javascript must be enabled to view this page.

members
magnitude
magnitudeUnassigned
count
unassigned
taxon
rank

428466\_out

3

superkingdom
2
3

phylum
201174
3

class
1760
3

85004
3
order

family
31953
3

genus
1678
3


SRS075878\_contig\_number\_10092SRS1041091\_contig\_number\_16497SRS150029\_contig\_number\_20454
species
3
1680
